# Rapidly Progressive Deep Polymicrobial Suprapubic Abscess Following Radical Inguinal Orchiectomy in a Patient With Poorly Controlled Diabetes

**DOI:** 10.7759/cureus.111101

**Published:** 2026-06-18

**Authors:** Jayanjali Bodavula, Adam D Long, Eric Delman

**Affiliations:** 1 College of Medicine, Kansas City University, Joplin, USA; 2 General Surgery, Mercy Hospital Pittsburg, Pittsburg, USA

**Keywords:** actinomyces israelii, embryonal cell carcinoma, esbl e.coli, polymicrobial soft tissue infection, post-operative complication, post-operative hematoma, radical inguinal orchiectomy, suprapubic abscess, testicular mass, uncontrolled diabetes mellitus

## Abstract

Radical inguinal orchiectomy is the gold standard surgical management for suspected testicular malignancy and is generally associated with low postoperative complication rates. Infectious complications are typically superficial and confined to the surgical incision or scrotal wall, while deep suprapubic or inguinal space abscess formation remains uncommon. We report a case of a 32-year-old man with poorly controlled type 2 diabetes mellitus who underwent a right radical inguinal orchiectomy for a testicular mass. On postoperative day 7, he developed mild inguinal swelling that was initially presumed to represent a benign hematoma. However, within days, he experienced worsening severe pain, erythema, leukocytosis, and systemic inflammatory response. Computed tomography of the abdomen and pelvis demonstrated a large loculated suprapubic fluid collection extending along the inguinal canal into the scrotum, concerning for a deep abscess. The patient underwent urgent incision and drainage with debridement and negative pressure wound therapy. Intraoperative cultures grew extended-spectrum-beta-lactamase-producing *Escherichia coli *and *Actinomyces israelii*, consistent with polymicrobial deep soft tissue infection. This case illustrates how seemingly benign postoperative changes may rapidly evolve into deep infection in high-risk patients, particularly in the setting of poorly controlled diabetes mellitus and hyperglycemia-associated impairment of host immune defenses. Furthermore, the anatomical continuity created by the inguinal approach may facilitate extension of infection beyond the superficial incision and along the spermatic cord tract. Radiographic findings may underestimate disease severity, and minimal gas does not exclude significant infection. Early operative source control and close clinical reassessment remain critical to preventing progression and optimizing outcomes in high-risk postoperative patients.

## Introduction

Testicular masses suspicious for malignancy are managed surgically, most commonly with radical inguinal orchiectomy. Although simple orchiectomy may be appropriate for benign conditions without concern for malignancy, radical inguinal orchiectomy remains the gold standard for suspected testicular cancer because it provides both diagnostic and therapeutic benefit while avoiding scrotal violation, which may disrupt lymphatic drainage and alter patterns of metastatic spread [1]. Radical inguinal orchiectomy is performed through an inguinal incision with high ligation of the spermatic cord at the level of the external inguinal ring, allowing removal of the testis and spermatic cord while minimizing tumor manipulation [1].

Radical inguinal orchiectomy is generally associated with low complication rates. Reported postoperative complications include pain, scrotal bruising, swelling, seroma, hematoma, and infection [1]. Overall, surgical site infection rates following scrotal and inguinal urologic procedures are low, and when infection occurs, it is most commonly superficial and confined to the surgical incision or scrotal wall. On the other hand, deep suprapubic or inguinal space abscess formation is an uncommon complication. Risk factors for postoperative infection include diabetes, obesity, immunosuppression, prolonged operative time, and scrotal violation during surgery [1]. Poorly controlled diabetes mellitus is a particularly important risk factor because hyperglycemia impairs neutrophil chemotaxis, phagocytosis, and oxidative burst activity [2]. Mechanistically, non-enzymatic glycosylation of cell surface receptors interferes with neutrophil response to chemoattractants, such as interleukin-8 (IL-8) and complement-component 5a (C5a) [3]. Hyperglycemia also inhibits nicotinamide adenine dinucleotide phosphate (NADPH) oxidase activity, reducing the generation of reactive oxygen species required for intracellular killing of phagocytized pathogens [4]. These alterations compromise host immune defense and wound healing.

Postoperative hematoma further increases infection risk by creating dead space and providing a nutrient-rich environment for bacterial proliferation [5]. In high-risk patients, postoperative swelling should therefore be monitored closely for signs of evolving infection. Deep space infections can also differ microbiologically from superficial surgical site infections. While superficial infections are typically caused by common skin flora such as *Staphylococcus aureus,* deep space infections may demonstrate polymicrobial involvement and include less commonly encountered organisms, particularly in high-risk hosts, such as those with uncontrolled diabetes [6].

In this report, we present a case of deep suprapubic abscess following radical inguinal orchiectomy in a patient with poorly controlled type 2 diabetes mellitus. Early postoperative swelling was presumed to represent a benign hematoma. However, the patient was ultimately found to have a polymicrobial deep suprapubic abscess with extension along the inguinal canal. This case highlights how seemingly benign postoperative changes may rapidly evolve into deep infection in high-risk patients and underscores the potential for atypical polymicrobial infection, anatomical spread along the spermatic cord tract, and the importance of early operative source control.

## Case presentation

A 32-year-old man with a history of insulin-dependent type 2 diabetes, hypertension, hyperlipidemia, and asthma presented to a community clinic with several months of progressively worsening right testicular swelling and pain. Scrotal ultrasonography demonstrated a 3.5 x 3.7 x 1 cm right testicle with heterogeneous echotexture involving the anterior aspect with preserved blood flow. The left testicle was normal in appearance. The heterogeneity of the right testicle was concerning for malignancy, and further evaluation was recommended.

He was referred to urology, where physical examination demonstrated a firm nodule in the anterior superior aspect of the right testicle. Initial serum tumor markers were within normal limits, with an alpha-fetoprotein of 2.5 ng/mL (reference <6.1 ng/mL) and a beta-human chorionic gonadotropin of <5 mIU/mL (reference <5 mIU/mL). Preoperative imaging demonstrated no significant intra-abdominal pathology. A right radical inguinal orchiectomy was performed. The procedure was completed via an inguinal approach with high ligation of the spermatic cord at the level of the external inguinal ring. The surgery proceeded without any complications and was well tolerated by the patient.

At postoperative day (POD) 7 follow-up, the patient reported mild swelling in the right inguinal area. A physical exam revealed a tender right inguinal mass that was clinically consistent with a hematoma without overlying erythema, ecchymosis, or drainage. The incision was clean, dry, and intact. Conservative observation of the hematoma was recommended.

However, by POD 10, the patient developed progressive right groin and suprapubic pain, worsening swelling, erythema, and malodorous red-brown purulent drainage from the incision site. He was started on trimethoprim-sulfamethoxazole for presumed cellulitis at the community clinic. Despite antibiotic therapy, his symptoms progressively worsened.

On POD 12, he presented to the emergency department with severe right groin pain rated 10/10, subjective fevers, chills, nausea, and dizziness. On admission, he was febrile to 101.2°F, hypertensive at 148/68 mmHg, and tachycardic with a heart rate of 108 beats per minute. Laboratory evaluation demonstrated leukocytosis (13.2 K/μL) and elevated inflammatory markers (C-reactive protein 233 mg/L and erythrocyte sedimentation rate 89 mm/Hr). He met the criteria for sepsis. Labs also showed hyperglycemia (serum glucose 240 mg/dL) with a hemoglobin A1c of 9.7%, consistent with poorly controlled diabetes mellitus (Table 1). On physical examination, the incision site was erythematous with active purulent drainage. There was marked tenderness and induration extending from the right inguinal region into the suprapubic area and scrotum, with a firm, fluctuant mass inferior to the incision. Computed tomography (CT) of the abdomen and pelvis with contrast revealed a large loculated fluid collection in the right groin and inguinal canal with minimal gas formation, concerning for a deep soft tissue suprapubic abscess (Figure 1).

**Table 1:** Key laboratory values at admission and during hospitalization following abscess drainage. Bpm: beats per minute; CRP: C-reactive protein; ESR: erythrocyte sedimentation rate; N/A: not available.

Parameter	Hospital day 1	Hospital day 2	Hospital day 3	Reference range
Temperature	101.2 °F	98.8 °F	97.9 °F	98.6 °F
Heart rate	108 bpm	89 bpm	92 bpm	60-100 bpm
Blood pressure	148/68 mmHg	125/78 mmHg	122/72 mmHg	120/80 mmHg
White blood cells	13.2 K/μL	11.8 K/μL	7.5 K/μL	4.3-11.0 K/μL
CRP	233 mg/L	N/A	N/A	<5 mg/L
ESR	89 mm/Hr	N/A	N/A	0-15 mm/Hr
Serum glucose	240 mg/dL	279 mg/dL	252 mg/dL	74-99 mm/dL
Hemoglobin A1c	9.7%	N/A	N/A	≤5.6%

**Figure 1:**
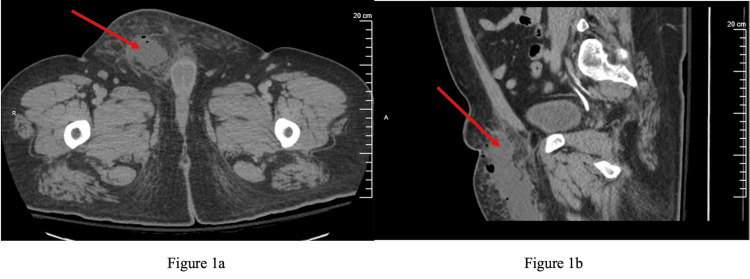
Contrast-enhanced computed tomography of the abdomen and pelvis demonstrating a large right suprapubic abscess extending into the inguinal canal (red arrows). (a) Axial view showing a loculated fluid collection with surrounding soft tissue stranding. (b) Sagittal view showing inferior extension of the abscess into the scrotum.

The patient was admitted and started on intravenous vancomycin and piperacillin-tazobactam antibiotics. General surgery was consulted, and the patient was taken to the operating room for incision and drainage of the abscess. Upon reopening the prior incision, copious purulent fluid was encountered, with approximately 200 mL evacuated. The abscess cavity tracked proximally within the inguinal canal and extended 10 cm distally into the scrotum, measuring 3.5 cm in depth. Given the extent of involvement, the incision was extended in a T-fashion to allow adequate exposure and drainage. Sharp and blunt debridement were performed, and minimal necrotic tissue was identified. The cavity was copiously irrigated, and a negative-pressure wound vacuum was then placed.

The intraoperative abscess culture grew extended-spectrum beta-lactamase (ESBL)-producing *Escherichia coli* and *Actinomyces israelii*. Blood cultures remained negative. Following incision and drainage, the patient demonstrated rapid clinical improvement with resolution of fever and leukocytosis. Despite insulin treatment, his blood glucose levels remained persistently elevated throughout the hospital stay (Table 1). He was discharged the following day in stable condition with a wound vacuum in place and oral amoxicillin-clavulanate antibiotics. At follow-up eight days after incision and drainage, the wound demonstrated healthy granulation tissue without any residual signs of infection. A timeline of events is described in Table 2.

Final pathology of the testicular mass revealed a 3.3 cm mixed germ cell tumor composed predominantly of embryonal carcinoma (>95%) and a minor yolk sac component. The tumor was confined to the testis, with rete testis and lymphovascular invasion present. The spermatic cord margin was negative for malignancy. Staging imaging revealed no metastatic disease. The treatment plan determined by oncology was to initiate bleomycin, etoposide, and cisplatin for three cycles. The patient continues to follow up with oncology for ongoing management.

**Table 2:** Postoperative clinical course and timeline of events. POD: postoperative day; ED: emergency department; CT: computed tomography; I&D: incision and drainage.

Postoperative day	Event
POD 0	Right radical inguinal orchiectomy.
POD 7	Mild swelling in the right inguinal area, presumed hematoma. The incision is clean, dry, and intact. Conservative observation recommended.
POD 10	Worsening right inguinal/scrotal pain, swelling, erythema, and malodorous red-brown purulent drainage from the incision site. Started on trimethoprim-sulfamethoxazole for presumed cellulitis.
POD 12	ED presentation with severe right scrotal pain rated 10/10, fever, chills, nausea, and dizziness. CT abdomen/pelvis showed deep suprapubic abscess. I&D performed.
POD 20 (Post-I&D day 8)	Incision healing well, wound demonstrated healthy granulation tissue, and no residual signs of infection present.

## Discussion

Radical inguinal orchiectomy is the standard surgical management for suspected testicular malignancy and is generally associated with low complication rates. Reported postoperative complications include pain, scrotal bruising, swelling, seroma, hematoma, and superficial wound infections [1, 7-9]. Surgical site infection rates following scrotal and inguinal urologic procedures have been reported between 1% and 3.5% [10]. When infection occurs, it is most commonly superficial and confined to the surgical incision or scrotal wall. Deep scrotal and testicular abscesses have been described in other clinical contexts, such as complicated epididymo-orchitis [11,12]. However, deep suprapubic and inguinal space abscesses with longitudinal tracking along the inguinal canal, as observed in our patient, are rarely described in the literature. Although radical inguinal orchiectomy is considered a clean operation, deep abscess formation may occur in the setting of postoperative hematoma or patient-specific risk factors such as poorly controlled diabetes [10].

Poorly controlled diabetes mellitus is a well-established independent risk factor for postoperative infectious complications. A systematic review and meta-analysis demonstrated that diabetes significantly increases the risk of surgical site infection across multiple surgical specialties [13]. Hyperglycemia impairs neutrophil chemotaxis and phagocytosis, reduces oxidative killing capacity, and contributes to microvascular dysfunction, collectively impairing wound healing and host immune defense [2,4,14]. In this case, the patient’s hemoglobin A1c of 9.7% and persistent hyperglycemia likely contributed to the rapid progression from a presumed postoperative hematoma to a deep space abscess. In fact, postoperative hematoma is a recognized risk factor for surgical site infection, as accumulated blood within a surgical bed creates dead space and provides a nutrient-rich environment that facilitates bacterial proliferation [15]. This risk may be further amplified in patients with poorly controlled diabetes. In our patient, although the localized swelling on POD7 was presumed to be a hematoma, the subsequent rapid progression to abscess formation suggests early evolving infection or secondary seeding of bacteria into the hematoma. In high-risk patients, early postoperative swelling may, therefore, mimic a benign hematoma yet rapidly evolve into a deep infection, warranting close monitoring and prompt reassessment with any clinical deterioration.

Additionally, the polymicrobial profile in our patient further underscores the severity of the infectious process. Intraoperative cultures grew ESBL *Escherichia coli* and *Actinomyces israelii*, reflecting a complex deep soft tissue infection rather than a typical superficial surgical site infection. While *Actinomyces* species are part of normal genitourinary flora, deep postoperative abscess due to *Actinomyces* has rarely been described. For example, an orchiectomy postoperative testicular abscess with *Actinomyces neuii* bacteremia has been reported as a unique case in literature, highlighting the unusual nature of such infections in the postoperative setting [16]. The presence of these organisms in a deep suprapubic abscess highlights the potential for atypical and aggressive polymicrobial infection in high-risk postoperative patients and reinforces the importance of early operative source control.

Furthermore, the anatomical considerations of the radical inguinal approach help explain the extent and distribution of infection observed in this case. Radical orchiectomy requires dissection through the inguinal canal with mobilization and high ligation of the spermatic cord, thereby establishing a potential contiguous space between the suprapubic region and the scrotum [1, 17]. Fascial continuity along this tract may permit longitudinal spread of fluid collections once infection is established [17]. In our patient, imaging demonstrated extension of the abscess along the inguinal canal into the scrotum, consistent with this anatomical pathway. While this operative approach is standard and generally safe, the resulting potential space may allow deep infection to propagate beyond the superficial incision, especially when host defenses are compromised, such as with poorly controlled diabetes.

Finally, radiographic findings must be interpreted in conjunction with the patient’s clinical presentation. CT of the abdomen and pelvis demonstrated a loculated fluid collection initially favored to represent a postoperative seroma or hematoma. However, the patient’s severe pain, systemic inflammatory response, and markedly elevated inflammatory markers raised concern for an evolving deep soft tissue infection. Additionally, minimal gas on imaging does not exclude significant infection, and radiographic findings may underestimate early disease severity. Given the risk of rapid progression in high-risk patients, including potential advancement to necrotizing soft tissue infection, prompt operative exploration was warranted. The patient’s rapid improvement following incision and drainage emphasizes that antibiotics alone are insufficient for loculated abscesses and reinforces early surgical source control as the cornerstone of management in deep postoperative infection.

## Conclusions

Deep suprapubic and inguinal space abscess following radical inguinal orchiectomy is an uncommon but potentially serious complication. In patients with poorly controlled diabetes, early postoperative swelling, presumed to represent a benign hematoma, may instead reflect evolving deep infection. Hyperglycemia-related immune dysfunction, combined with the presence of a postoperative hematoma, can facilitate rapid progression to a polymicrobial abscess. The anatomical continuity created by the inguinal approach may also permit longitudinal spread along the spermatic cord tract, resulting in extensive involvement beyond the superficial incision. Radiographic findings may underestimate disease severity, and minimal gas on imaging does not exclude significant soft tissue infection. Early operative source control and timely reassessment are therefore essential to prevent progression and optimize outcomes in high-risk postoperative patients.
